# Effects of ultrasonic vibration on the microstructure and mechanical properties of high alloying TiAl

**DOI:** 10.1038/srep41463

**Published:** 2017-01-24

**Authors:** Chen Ruirun, Zheng Deshuang, Ma Tengfei, Ding Hongsheng, Su Yanqing, Guo Jingjie, Fu Hengzhi

**Affiliations:** 1 School of Materials Science and Engineering, Harbin Institute of Technology, Harbin, 150001, China.

## Abstract

To modify the microstructure and enhance performances, the ultrasonic vibration is applied in the mould casting of TiAl alloy. The effects and mechanism of ultrasonic vibration on the solidifying microstructure and mechanical properties are investigated and the model for predicting lamellar colony size is established. After ultrasonic vibration, the coarse microstructure is well modified and lamellar colony is refined from 534 μm to 56 μm. Most of precipitated phases are dissolved into the lamellar colony leading to a homogenous element distribution. The phase ratio of **α**_**2**_-Ti_3_Al and **γ**-TiAl is increased, and the chemical composition is promoted to more close to equilibrium level by weakening the influence of **β**-alloying elements. The microhardness and yield strength are gradually improved by 23.72% and 181.88% due to the fine grain strengthening, while the compressive strength is enhanced by 24.47% through solution strengthening. The critical ultrasonic intensity (**I**_**b**_) for TiAl alloy is estimated at 220 W cm^−2^ and the model for average lamellar colony size is established as 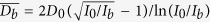. The ultrasonic refinement efficiency exponentially increases as the ultrasonic vibration time with a theoretic limit maximum value of ***E***_***lim***_ = 88% and the dominating refinement mechanism by ultrasonic vibration is the cavitation-enhanced nucleation rather than cavitation-induced dendrite fragmentation.

Since the thirty years of the last century, due to the high efficiency and simple adaptation, the ultrasonic technology has been widely applied in the biomedicine, chemistry and chemical engineering, and metallurgical industry^1^,^2^. The application of ultrasonic melt treatment in the light alloys has revealed broad prospects in the microstructure control and performance improvement for casting metal and alloys^3^. Wang Feng at al. has confirmed that the primary Al_3_Ti intermetallics in Al-0.4 wt%Ti alloy could be refined after ultrasonication and their morphology changed from large dendritic plate to small compact tablet; the significant refinement for ultrasonication is contributed by the enhanced heterogeneous nucleation^4^. Song Shangyu at al studied the distribution of SiC nanoparticles in magnesium melt obtaining a uniform dispersion of SiC nanoparticles and higher tensile properties^5^.

Despite of the abundant researches on the ultrasonic technology, the refinement mechanism of ultrasonic treatment is still controversial, and there are two prevailing theory for now - cavitation-enhanced nucleation and cavitation-induced dendrite fragmentation^2^,^4^. Due to opacity and non-visual observation, the effects of ultrasonic treatment on the crystallization behaviors have not established and the bone of contention is focused on the generation source of crystal nucleuses. In the cavitation-enhanced nucleation theory, the ultrasonic treatment could enlarge the melt supercooling and activate the inclusion, and the grain refinement is contributed by the enhanced heterogeneous nucleation^6^. In the cavitation-induced dendrite fragmentation mechanism, the ultrasonic treatment could generate powerful shock wave and jet stream, which will break the dendrite leading to the grain multiplication, and the fine grains are resulted from the dendrite fragmentation^7^.

Unfortunately, because of the cavitation erosion of radiator^8^ and low temperature requirement for transducer^9^, the ultrasonic technology is restricted in the application of high-temperature alloys, especially the high-reactivity alloys, such as the TiAl-based alloys^10^,^11^.

For now, as the high specific strength and excellent high temperature performances, the TiAl-based alloys have been successfully applied in the gas turbine engines to replace the heavy Ni-based superalloys, for which the weight reduction can be as much as 50%^12^,^13^,^14^. However, the casting of TiAl-based alloys presents coarse dendrite morphology and severely element segregation, which will severely deteriorate the mechanical performances and hinder back the industrial manufacture^15^. In general, the fine grains could remarkably improve the material strength and be beneficial for the elimination of element segregation and precipitates^16^. Therefore, it is of great significance to explore the application of ultrasonic vibration in TiAl-based alloys.

In this study, the ultrasonic vibration is implemented in the mould casting of Ti44Al6Nb1Cr2V alloy and the effects of ultrasonic vibration time on the microstructural characteristics and mechanical performances are elaborately investigated. Furthermore, a theoretical prediction model is established to estimate the average grain size and the ultrasonic refinement mechanism is also discussed.

## Results

### Effects of ultrasonic vibration on the macrostructure

**Figure 1:**
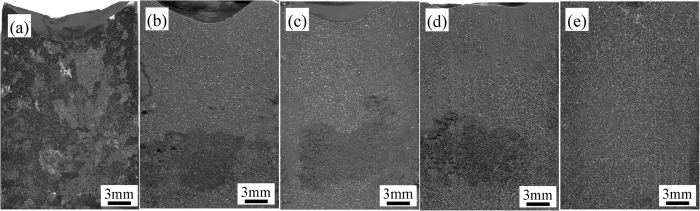
Macrostructure of Ti44Al6Nb1Cr2V alloy under different ultrasonic vibration time. (**a**) 0 s; (**b**) 15 s; (**c**) 30 s, (**d**) 45 s, (**e**) 60 s.

The macroscopic morphology of casting Ti44Al6Nb1Cr2V alloy is displayed in Fig. 1 after different ultrasonic vibration time. As shown in Fig. 1(a), without ultrasonic vibration, the specimen shows a typical casting macrostructure with coarse and inhomogeneous grains, in which the largest grain size is up to approximately 5 mm. Additionally, there is a big shrinkage cavity on the top of specimen. By contrast, when the alloy is imposed to ultrasonic vibration, the coarse casting macrostructure is transformed into uniform and fine macro-morphology, in spite of a smaller shrinkage cavity on the top and some shrinkage porosities in the bottom. Comparing with the Fig. 1(a), as the increasing of ultrasonic vibration time, it is obvious that the top shrinkage cavity and bottom shrinkage porosities gradually become smaller and finally the shrinkage cavity and porosity are almost entirely eliminated leading to a high quality casting ingot.

### Effects of ultrasonic vibration on the phase constitution

**Figure 2:**
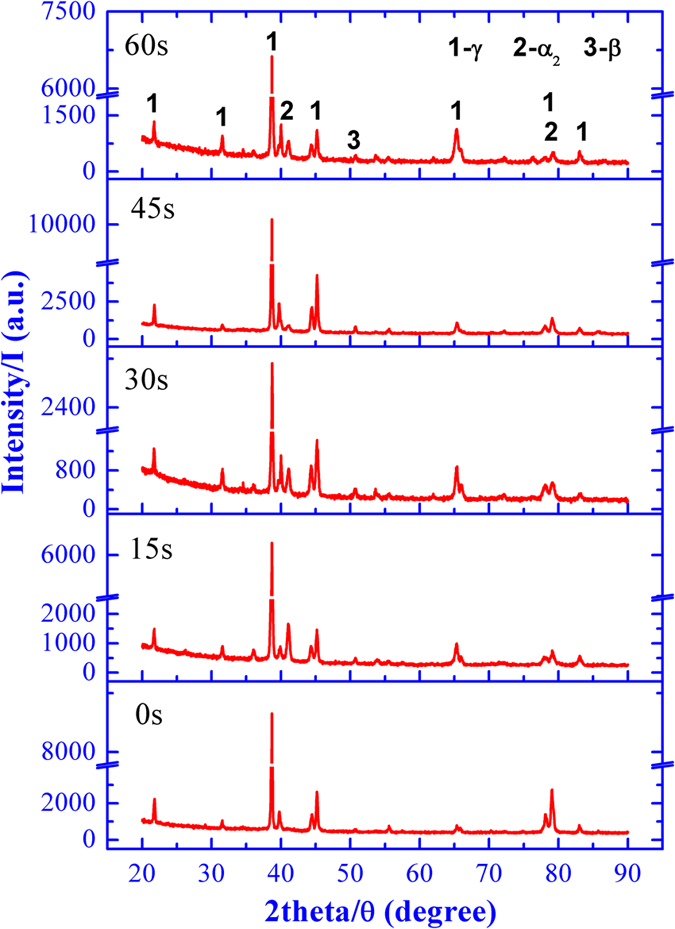
Effect of ultrasonic vibration on the phase constitution.

To investigate the effects of ultrasonic vibration time on the phase constitution of Ti44Al6Nb1Cr2V alloy, the phase constitution is analyzed by XRD, as shown in Fig. 2. According to the diffraction peak, all the specimens are consisted of **α**_**2**_-Ti_3_Al, **γ**-TiAl and **B**_**2**_ regardless of the ultrasonic vibration. However, as the change of diffraction intensity, the relative phase content is altered with the ultrasonic vibration time. Based on the diffraction theory^17^, the phase content (***ω***, wt.%) can be set as,


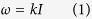


where the ***k*** is the proportionality factor and ***I*** is the integral under the strongest diffraction peak. The relative phase content is calculated by:


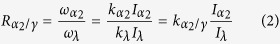



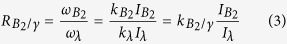


where the 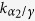 and 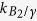 are the relative proportional coefficients.

**Figure 3:**
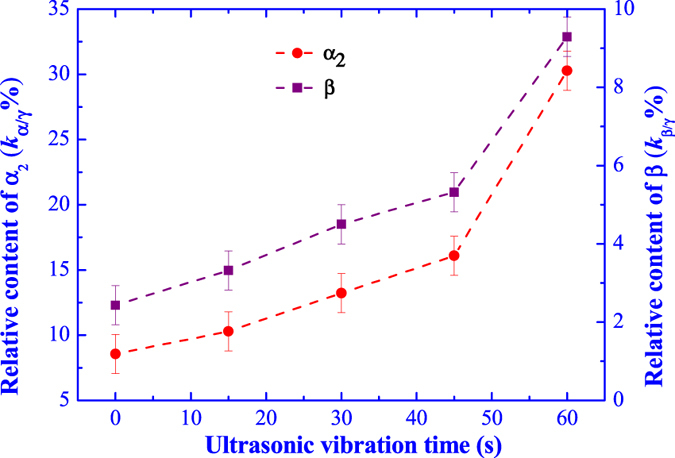
Effect of ultrasonic vibration time on the relative phase content.

Figure 3 illustrates the variation of relative phase contents with the ultrasonic vibration time, from which it can see that the **α**_**2**_-Ti_3_Al and **B**_**2**_ phases show an increasing relative phase contents with the ultrasonic vibration time. For Ti44Al6Nb1Cr2V alloy, the **α**_**2**_-Ti_3_Al and **γ**-TiAl phases are the major phases, while the **B**_**2**_ phase is the minor phase with a tiny content. Therefore, it can be inferred that the ultrasonic vibration could prompt the increasing of **α**_**2**_-Ti_3_Al phase and reducing of **γ**-TiAl phase, while the **B**_**2**_ phase content cannot be determined merely by the relative phase content and the variation of **B**_**2**_ phase content is confirmed by the microstructure observation in the following.

### Effects of ultrasonic vibration on the microstructure

**Figure 4:**
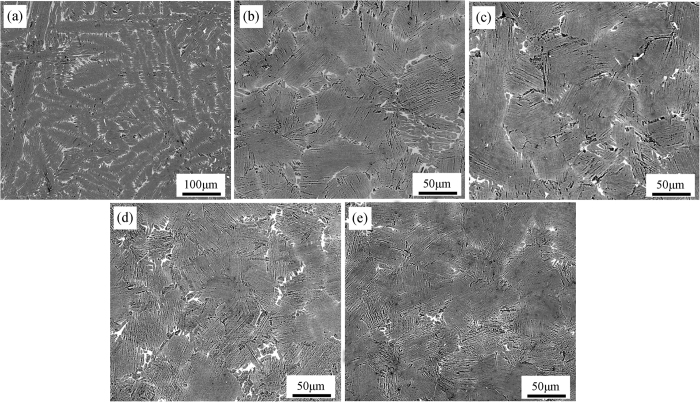
Microstructural characteristics of Ti44Al6Nb1Cr2V alloy under different ultrasonic vibration time. (**a**) 0 s; (**b**) 15 s; (**c**) 30 s, (**d**) 45 s, (**e**) 60 s.

The microstructural characteristics of TiAl alloy in absence and presence of ultrasonic vibration are displayed in Fig. 4, in which all the specimens present apparent full lamellar microstructure regardless of the ultrasonic vibration. The lamellar microstructure is consisted of **α**_**2**_-Ti_3_Al and **γ**-TiAl phases, which arrange alternatively forming the lamellar structure. For Ti44Al6Nb1Cr2V alloy in this study, due to the heavily alloying elements, it is inevitable to induce element segregation and precipitated phases (here collectively called **B**_**2**_ phase for convenient expression)^18^. As shown in Fig. 4(a), without ultrasonic vibration, the specimen shows coarse lamellar colony with severely element segregation, in which large quantity of white **B**_**2**_ phases distributed at the lamellar colony boundary as well as in the lamellar colony. However, as the increasing of ultrasonic vibration time, the coarse lamellar colony is significantly refined and the abundant of white **B**_**2**_ phases are gradually eliminated. Majority of remnant **B**_**2**_ phases locate at the lamellar colony boundary and the element segregation in the lamellar colony entirely disappears.

**Figure 5:**
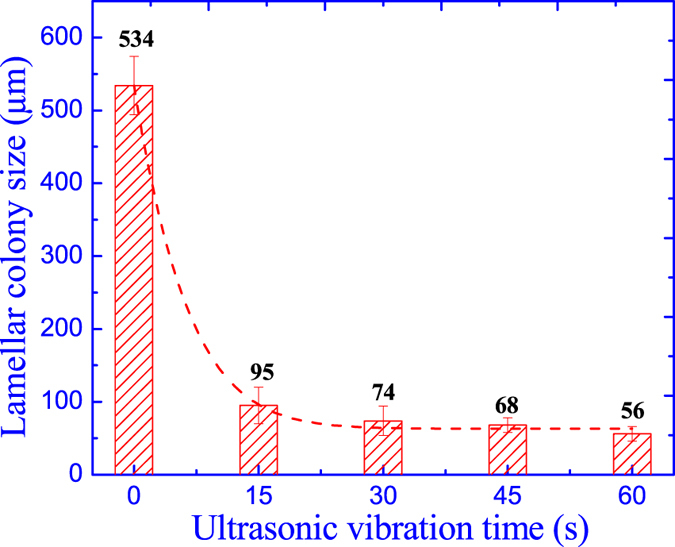
Effect of ultrasonic vibration time on the lamellar colony size.

The variation of lamellar colony size as the ultrasonic vibration time is illustrated in Fig. 5, in which the lamellar colony is significantly refined after the ultrasonic vibration. For the comparing specimen without ultrasonic vibration, the coarse microstructure shows large lamellar colony size up to 534 μm, while the lamellar colony size is significantly refined to 95 μm after ultrasonic vibration for 15 s. Besides, with the further increasing of ultrasonic vibration time, the lamellar colony is gradually decreased with a small reduction and the minimum lamellar colony size is only 56 μm after ultrasonic vibration for 60 s.

### Effects of ultrasonic vibration on the precipitated phase

**Figure 6:**
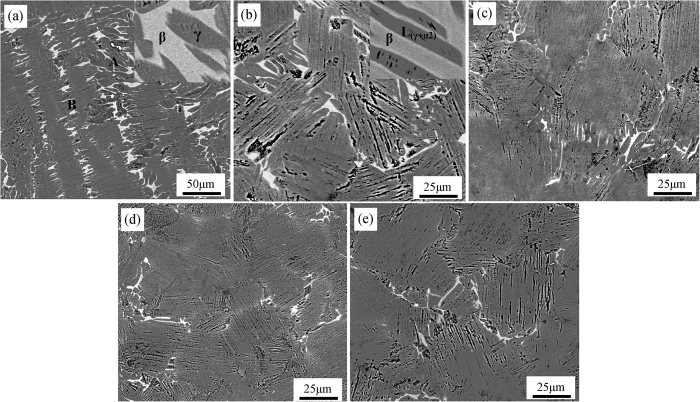
Effects of ultrasonic vibration time on the morphology and distribution of precipitated phases (**B**_**2**_). (**a**) 0 s; (**b**) 15 s; (**c**) 30 s, (**d**) 45 s, (**e**) 60 s.

Due to the severe element segregation, there forms larger quantity of precipitated phases (**B**_**2**_) and the morphology and distribution of **B**_**2**_ phases are displayed in Fig. 6. Due to severer element segregation without the ultrasonic vibration, there is a vast amount of large bulk precipitated phases (**B**_**2**_) located in the lamellar colony as well as at the lamellar colony boundary. In the lamellar colony, the large bulk precipitated phases (**B**_**2**_) form narrow banded distribution marked by **B** in Fig. 6(a). Furthermore, there are some block **γ**-TiAl phases mixed with the bulk precipitated phases (**B**_**2**_) displayed as the inserted illustration.

**Figure 7:**
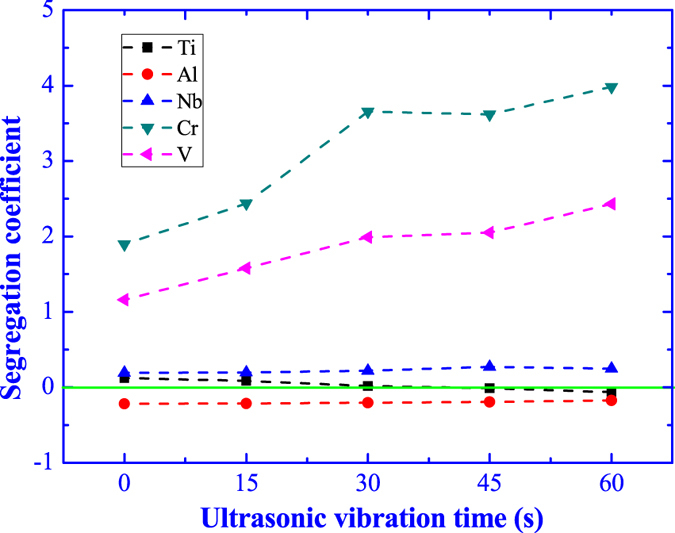
Effect of ultrasonic vibration time on the element segregation coefficient.

By contrast, after the ultrasonic vibration for 15 s, there are only small bulk precipitated phases (**B**_**2**_) continuous distributed at the lamellar colony boundary, while the narrow banded precipitated phases (**B**_**2**_) in the lamellar colony almost disappear. The precipitated phases (**B**_**2**_) plug into the lamellar colony (**L**_(**γ+α2**)_) forming interlock boundary structure and no block **γ**-TiAl phases coexist with the precipitated phases (**B**_**2**_) shown as the inserted illustration in Fig. 7(b). As the increasing of ultrasonic vibration time, the precipitated phases (**B**_**2**_) gradually become less and smaller, and majority of the remnant precipitated phases (**B**_**2**_) are located in the triangular domain instead of the network continuous distribution along the lamellar colony boundary. After ultrasonic vibration for 60 s, most of precipitated phases (**B**_**2**_) are eliminated by dissolving into lamellar colony forming a homogeneous fully lamellar microstructure.

By contrast, after the ultrasonic vibration for 15 s, there are only small bulk precipitated phases (**B**_**2**_) continuous distributed at the lamellar colony boundary, while the narrow banded precipitated phases (**B**_**2**_) in the lamellar colony almost disappear. The precipitated phases (**B**_**2**_) plug into the lamellar colony (**L**_(**γ+α2**)_) forming interlock boundary structure and no block **γ**-TiAl phases coexist with the precipitated phases (**B**_**2**_) shown as the inserted illustration in Fig. 7(b). As the increasing of ultrasonic vibration time, the precipitated phases (**B**_**2**_) gradually become less and smaller, and majority of the remnant precipitated phases (**B**_**2**_) are located in the triangular domain instead of the network continuous distribution along the lamellar colony boundary. After ultrasonic vibration for 60 s, most of precipitated phases (**B**_**2**_) are eliminated by dissolving into lamellar colony forming a homogeneous fully lamellar microstructure.

The chemical compositions of the matrix (lamellar colony) and precipitated phases (**B**_**2**_) are measured by the energy spectrum analysis in scanning electrochemical microscopy and the statistical average compositions are listed in Table 1. Due to the severer element segregation and large quantity of precipitated phases (**B**_**2**_), the matrix without ultrasonic vibration shows a big deviation from the nominal alloying composition. The heavy metal elements (Ti, Nb, Cr and V) are segregated in the precipitated phases (**B**_**2**_), while the light metal element (Al) is enriched in the matrix. As the alleviation of element segregation and elimination of precipitated phases (**B**_**2**_) by ultrasonic vibration, more heavy alloying elements are redistributed into the matrix and the chemical composition becomes more close to the nominal alloying composition.

On the other side, while the precipitated phases (**B**_**2**_) become less as the ultrasonic vibration time, more alloying elements are accumulated in the precipitated phases (**B**_**2**_). In this work, the extent of element segregation in the precipitated phases (**B**_**2**_) is evaluated by the element segregation coefficient (***K***_***s***_), which is defined as,


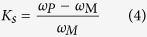


where the ***ω***_***M***_ and ***ω***_***P***_are the content of alloying element in the matrix and precipitated phases (**B**_**2**_), respectively. The plus and minus sign symbols represent the positive segregation (***K***_***s***_ > 0) and negative segregation (***K***_***s***_ < 0), respectively; the absolute value of segregation coefficient indicates the extent of element segregation and the bigger ***K***_***s***_, the severer element segregation. According to the chemical compositions in Table 1, the element segregation coefficient is counted and the results are illustrated in Fig. 7.

The Al element shows a small negative segregation and the segregation coefficient gradually decrease with a tiny reduction as the ultrasonic vibration time. The Ti element presents a slight positive segregation and the segregation coefficient is almost constant regardless of the ultrasonic vibration time. The Nb element appears a mild positive segregation with a small increment as the ultrasonic vibration time. The Cr and V elements lead to a severe positive segregation and the segregation coefficient is remarkable increased as the increasing of ultrasonic vibration time. After ultrasonic vibration for 60 s, the contents of Cr and V in the precipitated phases (**B**_**2**_) increase up to 5.23% and 7.07%, respectively, much higher than the normal alloying constitution (1% Cr and 2% V).

### Effects of ultrasonic vibration on the mechanical properties

**Figure 8:**
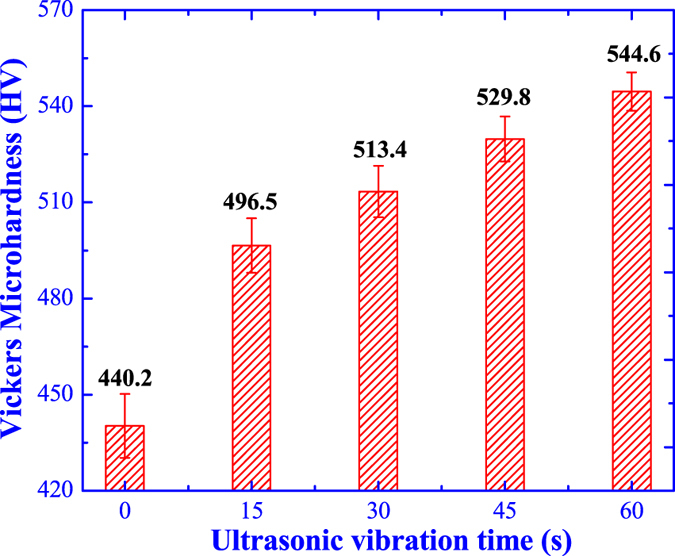
Effect of ultrasonic vibration on the Vickers microhardness.

As the microstructure modification and lamellar colony refinement by the ultrasonic vibration, the mechanical performances must have been significantly affected. The microhardness and compressive properties are measured for evaluating the effects of ultrasonic vibration on mechanical performances of Ti44Al6Nb1Cr2V alloy. Figure 8 displays the variation of Vickers microhardness as the ultrasonic vibration time and it is obvious that the microhardness is gradually improved as the increasing of ultrasonic vibration time. The initial microhardness without ultrasonic vibration is only 440.2HV, while the microhardness is increased to 544.6HV higher by 23.72% after ultrasonic vibration for 60 s.

**Figure 9:**
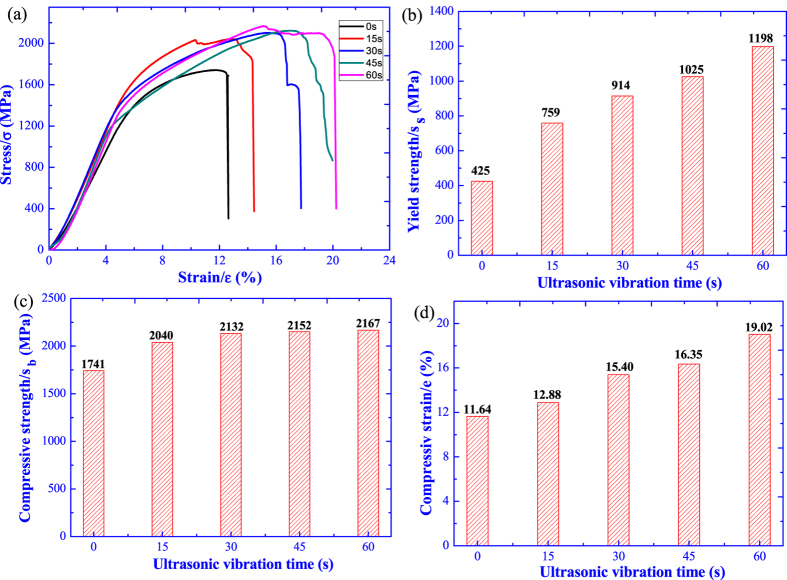
Compressive properties of Ti44Al6Nb1Cr2V alloy under different ultrasonic vibration time. (**a**) stress-strain curve; (**b**) yield strength; (**c**) compressive strength; (**d**) compressive strain.

The compressive properties of Ti44Al6Nb1Cr2V alloy under ultrasonic vibration are tested at room temperature and the stress-strain curve, yield strength, compressive strength and strain (here refer to the ultimate stress and strain at the fracture failure) are shown in Fig. 9. Regardless of the ultrasonic vibration, all the testing specimens exhibit abrupt fracture failure with a high stress and low strain. In the absence of ultrasonic vibration, the Ti44Al6Nb1Cr2V alloy shows poor compressive properties, and the yield strength, compressive strength and strain are only 425 MPa, 1741 MPa and 11.64%, respectively. After ultrasonic vibration for 15 s, all compressive properties are improved. However, with further increasing of ultrasonic vibration time, the yield strength and compressive strain are gradually improved, while the compressive strength is almost constant with a tiny increment. After ultrasonic vibration for 60 s, the yield strength is increased to 1198 MPa added by 181.88%, the compressive strength reaches up to 2167 MPa enhanced by 24.47% and the compressive strain is improved to 19.02% enlarged by 63.4%.

### Effects of ultrasonic vibration on the fracture morphology

**Figure 10:**
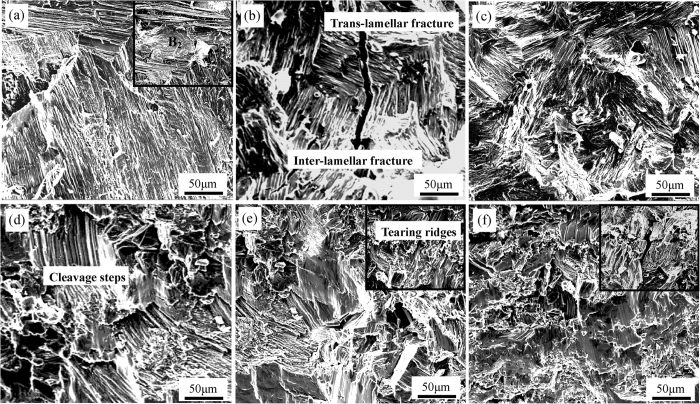
Compressive fracture morphology under different ultrasonic vibration time. (**a**) and (**b**) 0 s; (**c**) 15 s; (**d**) 30 s, (**e**) 45 s, (**f**) 60 s.

The compressive fracture morphology of Ti44Al6Nb1Cr2V alloy after ultrasonic vibration is displayed in Fig. 10, in which all the testing specimens appear typical brittle fracture characteristics regardless of ultrasonic vibration. Due to the coarse lamellar colony without ultrasonic vibration, the Ti44Al6Nb1Cr2V alloy exhibits the mixed inter-lamellar and trans-lamellar fracture (see Fig. 10(b)). Additionally, as the brittle phase, the precipitated phases (**B**_**2**_) are prone to result in large stress concentration leading to crack initiation and fracture failure, as shown in the inserted illustration of Fig. 10(a). After ultrasonic vibration for 15 s, although the coarse lamellar colony has been well refined, the fracture morphology shows no obvious change, as displayed in Fig. 10(c). Under ultrasonic vibration time for 30 s, the fracture morphology shows inter-lamellar cleavage fracture features forming abundant of cleavage steps (see Fig. 10 (d)). As further increasing of ultrasonic vibration time, besides the cleavage fracture, the fracture presents vast amount of tearing ridges at the lamellar colony boundary (see the inserted illustration in Fig. 10(e)). The refined precipitated phases (**B**_**2**_) could prevent the crack growth and change the crack propagation direction, and the crack will bypass the precipitated phases (**B**_**2**_) leading to shedding of small particles from the lamellar colony boundary, as displayed in the inserted illustration of Fig. 10(f).

## Discussions

When the ultrasound wave propagates into the melt, there will form three prime phenomena - ultrasonic cavitation, acoustic streaming and acoustic radiation pressure^2^. Due to the ultrasonic cavitation effect, it could generate innumerable micro hot-spot during the collapse of cavitation, in which the transient temperature is up to 5000 K and the pressure is as high as 5 GPa^19^,^20^,^21^. The acoustic streaming could significantly accelerate the melt flow higher 10 times of the thermal flow, which will remarkably enhance the heat transfer and solute exchange leading to a homogeneous temperature and solute field^22^. As the mechanical wave, the ultrasonic vibration must cause a pressure gradient leading to an additional static head, which could have significant effects on the melt flow and feeding.

Generally, for certain melt, there is a threshold value of ultrasonic intensity (***I***), which is the least energy required for full developed ultrasonic cavitation^2^ and only above the threshold value the ultrasonic vibration could effectively affect the melt. The ultrasonic intensity (***I***) is defined as the following form refs 23 and 24,


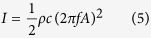


Where ***ρ*** is the density of propagation medium, ***c*** is the speed of ultrasound in the propagation medium, ***f*** is the ultrasonic frequency, and ***A*** is the ultrasonic amplitude. In this work, the maximum available ultrasonic amplitude at the radiating face is about 5~7 μm. Assuming the molten melt of Ti44Al6Nb1Cr2V alloy **ρ** = 4000 kg m^−3^ and **c** = 1500 m s^−1^, so the nominal maximum ultrasonic intensity is about 1000 W cm^−2^. According to the previous study^2^, the threshold value of ultrasonic intensity in metals and alloys is about 80~100 W cm^−2^, so the ultrasonic vibration could effectively influence the molten melt in this study. As discussion above, the ultrasonic vibration has obviously refined the coarse lamellar colony, reduced the element segregation and eliminated the precipitated phases (**B**_**2**_). After ultrasonic vibration, the coarse microstructure is well modified into a homogenous fine grain structure, and the microhardness and compressive properties are substantially improved as well.

In the free solidification without ultrasonic vibration, because of the heat absorption and lateral heat dissipation of the ceramic mould, the casting will progressively solidify from the surface to the centre leaving a cavity in the middle-upper part; due to the vast solidification contraction of TiAl-based alloys, there is not enough molten melt to feed the cavity, so there forms a big shrinkage cavity on the top of specimen. By contrast, under ultrasonic vibration, because of the homogeneous temperature and solute field by acoustic streaming, the casting is prone to volume solidification, which is beneficial to eliminate the shrinkage cavity. However, the volume solidification has a big tendency to cause dispersed shrinkage porosity. The forming criterion of shrinkage porosity is,


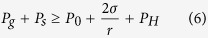


where the ***P***_***g***_ is the pressure of gas evolution, ***P***_***s***_ is the feeding resistance, ***P***_***0***_ is the atmospheric pressure, ***σ*** is the interfacial tension, ***r*** is the cavity radius and ***P***_***H***_ is the metal static head. For a certain solidification process, the P_***g***,_ P_***0***_, ***P***_***H***_and ***σ***retain constant; the ***P***_***s***_ is related to the dendrite morphology and grain size; the ***r*** can be significantly affected by the gas content. After ultrasonic vibration, the coarse microstructure is modified into fine non-dendrite grain structure, which is conduce to avoid the blocking of intergranular feeding channel and decrease the feeding distance, so the ultrasonic vibration could reduce the feeding resistance (***P***_***s***_). Due to the degassing effect of ultrasonic vibration^25^, the cavity radius (**r**) will decease leading to a higher pressure resistance. Furthermore, the acoustic radiation pressure will generate an additional static head (***P***_***A***_), so the Eq. 6 is adapted to,


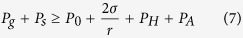


And the additional static head (***P***_***A***_) can be calculated by,


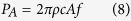


According as the Eq. 7, it can reckon that the maximum ***P***_***A***_ = 2.77 × 10^6^ Pa as high as 20 times of the atmospheric pressure (***P***_***0***_), which would reduce the forming tendency of shrinkage porosity. Summarizing the above discussions, the ultrasonic vibration could effective eliminate the shrinkage cavity and porosity obtaining a high quality casting ingot.

Although abundant of researches have certified the high-efficiency grain refinement and morphology modification by ultrasonic vibration, there have great controversies on the refinement mechanism for lack of theoretical foundation and visualized experimental observation^2^,^4^,^6^. For now, the most extensive mechanism is the cavitation-enhanced nucleation theory based on the ultrasonic cavitation effect, in which the grain refinement efficient is depended on the ultrasonic intensity and the higher intensity, the refiner grain.

When the ultrasonic vibration propagates through the melt, the ultrasonic intensity and amplitude will diminish or attenuate with the propagation distance due to the absorption and reflection of melt^23^. The attenuation of ultrasonic intensity (***I***) as the propagation distance (***x***) is described by,


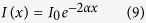


where the***I***_***0***_ is the initial ultrasonic intensity and ***α*** is the attenuation coefficient. Combining the Eqs 5 and 9, it can be deduced,


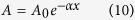


where the ***A***_***0***_ is the initial ultrasonic amplitude. Previous research by Ma qian *et al*.^23^ reveals that the dependence of grain density (***G***) on ultrasonic amplitude (***A***) is fundamentally analogous along the propagation direction and the attenuation of the ultrasonic amplitude (***A***) with the propagation distance can be assessed according to the variations of grain density (***G***) with the propagation distance. So the grain density (***G***) as the propagation distance can be formed by,


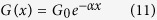


where is ***G***_***0***_ the initial grain density. As the grain size (***D***) shows a reciprocal relation with the grain density (***G***), therefore, the distribution of grain size along the ultrasonic propagation distance obeys the following equation,


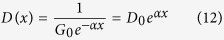


where ***D***_***0***_ is the initial grain size. Therefore, the grain size should exponentially decrease as the propagation distance. However, in our study, the grain size difference (here refer to the lamellar colony size) along the specimen from the top to the bottom is very tiny and the grain size is given as the statistical average grain size.

In theory, the average grain size 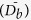 after ultrasonic refinement can be calculated by,


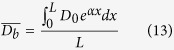


where the***L***is the propagation distance at the critical ultrasonic intensity (***I***_***b***_, threshold value). Considering of the homogenous temperature field by ultrasonic vibration, it is reasoned to assume that the attenuation coefficient (***α***) is constant along the ultrasonic propagation direction,


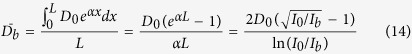


where the *αL* = ln(*I*_0_/*I*_*b*_)/2 based on the Eq. 9. As indicating by the Eq. 14, the theoretic average grain size is only determined by the initial grain size and ultrasonic intensity.

**Figure 11:**
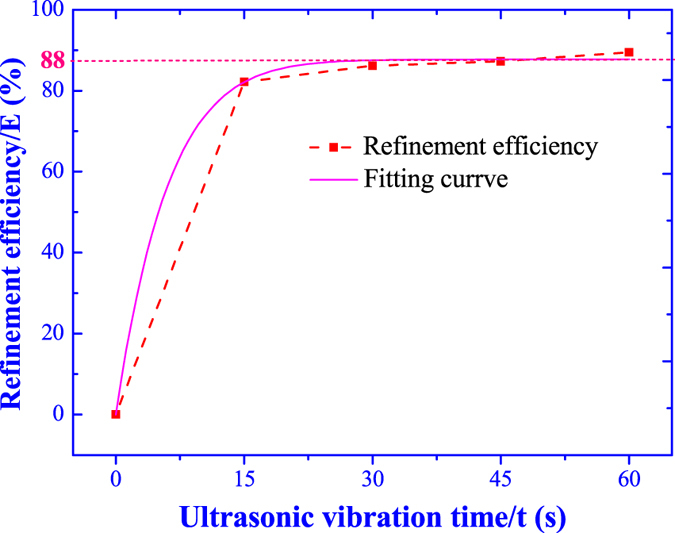
Grain refinement efficiency under different ultrasonic vibration time.

In our study, the grain refinement efficiency (***E***) as the ultrasonic vibration time (***t***) is defined as,


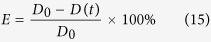


where the ***D**(**t***) is the refind grain size under different ultrasonic vibration time. The variation of grain refinement efficiency as the ultrasonic vibration time is shown in Fig. 11. The grain refinement efficiency is increased as the increasing of ultrasonic vibration time and the grain refinement efficiency is remarkably improved to 82.2% in the first 15 s. However, as further increasing of ultrasonic vibration time, the grain refinement efficiency retains constant with a tiny increment. So, the grain refinement efficiency of ultrasonic vibration is not much relevant with the treatment time and the grain refinement efficiency could reach the peak value within a very short time.

The fitting curve of grain refinement efficiency as the ultrasonic vibration time is displayed in Fig. 11 and the fitting formula is optimized as,


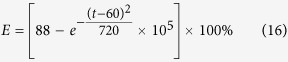


According to the Eq. 16, the grain refinement efficiency exponentially increases as the ultrasonic vibration time and there is a theoretic limit maximum grain refinement efficiency ***E***_***lim***_ = 88%.

As a corollary, it can deduce the theoretic average grain size 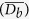 based on the Eq. 15,


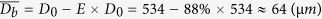


Similarly, combining the Eq. 14, the theoretical critical ultrasonic intensity (***I***_***b***_) can be concluded,


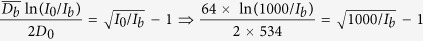


This is a complex function and the eigenvalue is evaluated by the graphic analytic method, in which the ***I***_***b***_ is reckoned about 120 W cm^−2^ much higher than the light alloys (80~100 W cm^−2^).

The formation, growth, expansion and collapse of one cavitation bubble only continues for several cycle periods and the collapse of one cavitation bubble will split into several micro-bubbles, which will become new cavitation bubbles in chain reaction^2^. Due to the high ultrasonic frequency up to 20 kHz, once the high-intensity ultrasonic vibration propagates in the melt, there will instantaneously generate countless cavitation bubbles by chain reaction in several milliseconds. As results, the grain size could be significantly refined in a vary short time of ultrasonic vibration (15 s), as shown in Fig. 5 and the grain refinement efficiency nearly retains constant for further increasing of ultrasonic vibration time, as demonstrated in Fig. 11.

By contrary, the rapid grain refinement efficiency presents a big contradiction with the cavitation-induced dendrite fragmentation theory^26^, which is another prevailing ultrasonic refinement mechanism. In the cavitation-induced dendrite fragmentation theory, the ultrasonic vibration could break the dendrites and the grain refinement is contributed by the dendrite multiplication. However, at the initial solidification, there is no dendritic crystals and it is impossible to induce obvious grain refinement in a vary short time by dendrite fragmentation. Synthesizing the above analysis, the cavitation-induced dendrite fragmentation theory could not be or at least not the dominate mechanism for the ultrasonic refinement in TiAl-based alloys.

As the **β**-alloying elements (Nb, Cr, V) could extend the **β** phase region, the equivalent Ti-Al binary phase diagram will deviate from the equilibrium diagram to higher Al content, which will lead to higher phase ratio of **α**_**2**_-Ti_3_Al and **γ**-TiAl theoretically^27^,^28^. However, after ultrasonic vibration, the phase ratio of **α**_**2**_-Ti_3_Al and **γ**-TiAl is gradually increased as the ultrasonic vibration time, implying the equivalent Ti-Al binary diagram returns back close to the equilibrium diagram. It can be inferred that the ultrasonic vibration could weaken the effects of **β**-alloying elements on the equilibrium diagram and promote the solidification more close to the equilibrium solidification.

As the uniform temperature and solute field by acoustic streaming, all the crystals form and grow in the homogeneous melt, which is beneficial for the decrease of element segregation. Additionally, due to the finer grain size and higher melt flow after ultrasonic vibration, the solute diffusion distance is obviously reduced and the diffusion coefficient is apparently increased at the same time, therefore the solute concentration difference becomes smaller leading to an even element distribution.

**Figure 12:**
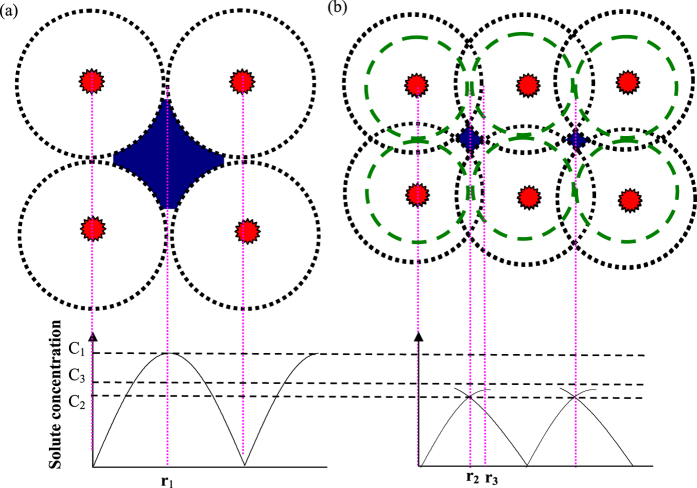
Effect of ultrasonic vibration on the solute enrichment at the crystal growing interface. (**a**) without ultrasonic vibration; (**b**) under ultrasonic vibration.

Without ultrasonic vibration, as the coarse lamellar colony, there are large quantity of residual spaces between the grain boundaries (see Fig. 12) resulting in more residual melt, so there forms vast abundant of large bulk precipitated phases (**B**_**2**_) at the grain boundary. With the grain refinement under ultrasonic vibration, most of bulk precipitated phases (**B**_**2**_) are gradually dissolved into lamellar colony and more alloying elements are redistributed into lamellar colony resulting a alloying composition more close to the equilibrium level.

In spite of elimination of precipitated phases (**B**_**2**_) by ultrasonic vibration, the alloying element segregation in the precipitated phases (**B**_**2**_) becomes severer as the ultrasonic vibration time, as illustrated in Fig. 7. In the constituent supercooling theory^29^, the solute is gradually enriched at the crystal growing interface by solute redistribution during the crystal growth process. There is a critical solute concentration, which is the minimum solute concentration necessary for constituent supercooling inducing new crystal nucleus. When the solute concentration reaches the critical solute concentration, the crystal will stop growing forming new crystal nucleus. In the free solidification without ultrasonic vibration, due to the uniform competitive crystal growth, there is no solute field overlap (**r**_**1**_) between the adjacent crystals and the solute concentration reaches the critical concentration (**C**_**1**_) at the grain boundary^30^, as shown in Fig. 12(a).

In the ultrasonic cavitation theory, there will generate high pressure up to 5 GPa in the micro hot-spot during the collapse of cavitation. The crystallization temperature (***T***_***P***_) and the pressure (***P***) obey the Clausius-Clapeyron equation,


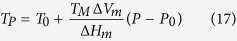


Where ***T***_***0***_ is the liquidus temperature at the atmospheric pressure (***P***_***0***_), ***∆V***_***m***_ and ***∆H***_***m***_ are the molar volume change and molar enthalpy change during melting process, respectively. Therefore, it can be reckoned the high pressure induced by ultrasonic cavitation must substantially improve the crystallization temperature, which is equivalent to improve the thermal supercooling. In consequence, the solute field will be enlarged from the constituent supercooling region (**r**_**2**_) to the ultrasonic region (**r**_**3**_) leading to an overlap region of solute enrichment, as displayed in Fig. 12(b). Thereby, after ultrasonic vibration, the solute enrichment is overlapped at the grain boundary leading to severer element segregation in the precipitated phases (**B**_**2**_). With the grain refinement as the ultrasonic vibration time, the constituent supercooling region (**r**_**2**_) becomes smaller and the overlap region (**r**_**3**_) further enlarge, so the element segregation coefficient gradually increases with the ultrasonic vibration time. On another side, the Cr and V elements show a higher partition coefficient resulting in higher element enrichment during the solute redistribution, so the Cr and V present larger segregation coefficient.

**Figure 13:**
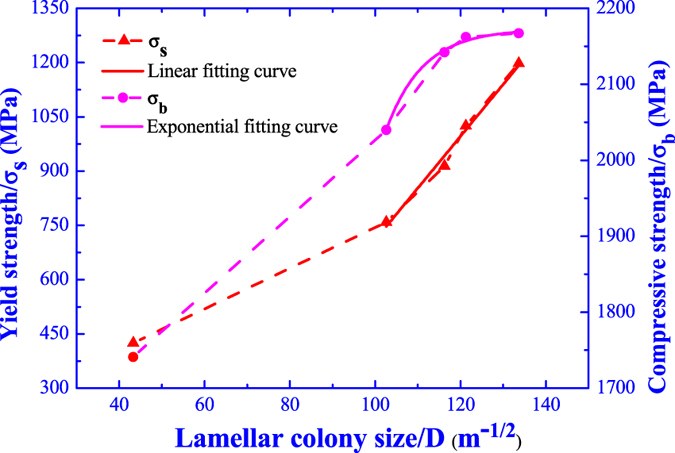
Variation of yield strength and compressive strength with the lamellar colony size.

In the fine grain strengthening theory, the interface hinders the dislocation motion forming dislocation pile-ups and prevents the crack propagation, and the strengthening effect is depended on the amount of grain boundary^31^. As the grain refinement, the dislocation multiplication and crack propagation are dispersed located at the more grain boundaries, which is beneficial for reducing the internal stress concentration and improving the integral plastic deformation. For eliminating the uncertainty factors and identifying the influence of ultrasonic vibration time on the performances, the specimen without ultrasonic vibration is not be considered in the following discussions for strength. In this study, the variation of lamellar colony size with the yield strength and compressive strength are illustrated in Fig. 13 and both the yield strength and compressive strength are increased as the decrease of lamellar colony size.

According to the Hall-Petch equation, there is a linear dependence between the material strength (***σ***) and grain size (***D***),


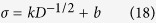


Where the ***k*** and ***b*** are the constants. The fitting curve for yield strength and compressive strength with the lamellar colony size are illustrated in Fig. 13. It is obvious that the yield strength and lamellar colony size obey the Hall-Petch equation, while the compressive strength shows an exponential relationship with the lamellar colony size. Therefore, under ultrasonic vibration, the yield strength enhancement is contributed by the fine grain strengthening and there is another strengthening mechanism for the compressive strength improvement.

Besides of the grain refinement, the ultrasonic vibration has alleviated the element segregation, eliminated the large bulk precipitated phases (**B**_**2**_) and promoted more alloying elements uniformly distributed in the lamellar colony. The small precipitated particles (**B**_**2**_) could decrease the stress concentration reducing the crack initiation and the more alloying elements will improve the solution strengthening, which are the main contribution to the compressive strength improvement. On the other side, as the higher strength of **γ**-TiAl phase, the decreasing of **γ**-TiAl phases will cut down the strength. In conclusion, the constant compressive strength with the ultrasonic vibration time is the competition of **γ**-TiAl phases reduction and solution strengthening.

**Figure 14:**
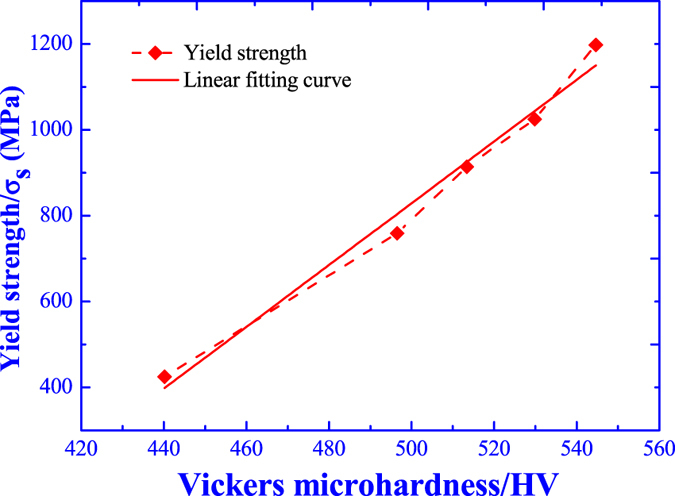
Dependence of yield strength on the microhardness.

Figure 14 illustrates the dependence of yield strength on the microhardness, and the yield strength is apparently improved as the increasing of microhardness, which shows a linear relation as indicated by the linear fitting curve. Hence, it is reasonable to deduce the increase of microhardness resulting from the fine grain strengthening by ultrasonic vibration as well as the yield strength enhancement.

In the micro-plastic deformation, the main deformation is the elastic deformation with a tiny plastic deformation by the dislocation motion and the specimens are subjected to the micro-plastic deformation during the testing of microhardness and yield strength. Most of the dislocation motions are blocked by the grain boundary leading to the reinforcement effects^32^,^33^. Consequently, the microhardness increment and yield strength enhancement are contributed by the ultrasonic grain refinement strengthening.

During the compressive fracture process, the specimen is undergone macro-plastic deformation and the dominant deformation mechanism is boundary sliding accompanied by intragranular dislocations slip and deformation twins, so the compressive strength is the results of interact competition between the softening of grain interiors and hardening of grain boundaries^34^,^35^.

**Figure 15:**
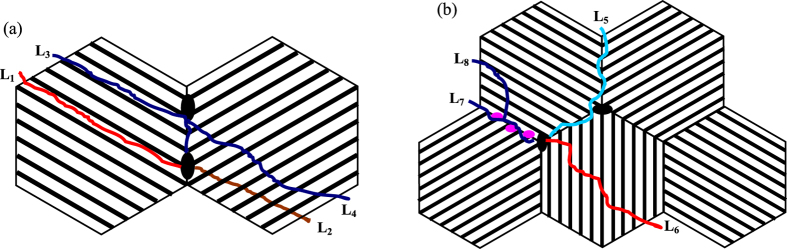
Schematic diagram of lamellar colony fracture: (**a**) without ultrasonic vibration; (**b**) under ultrasonic vibration.

With the increasing of macro-plastic deformation, more dislocation pile-ups are gathered at the precipitated phase on the grain boundary, which will cause a high stress concentration inducing the crack initiation, and the cracks propagate along or across the lamellar forming inter-lamellar fracture (**L**_**1**_) and trans-lamellar fracture (**L**_**2**_), as shown in Fig. 15(a). The large bulk precipitated phases (**B**_**2**_) could prevent the crack growth and turn the propagation direction forming inter-lamellar fracture (**L**_**3**_) or trans-lamellar fracture (**L**_**4**_). Considering the intrinsic characteristics of intermetallic compounds, it is difficult for dislocation slip and deformation twins in **α**_**2**_-Ti_3_Al and **γ**-TiAl crystals under room temperature^36^, so the macro-plastic deformation is crucially depended by the boundary sliding.

Under ultrasonic vibration, due to the more solution strengthening, it becomes more difficult for the dislocations slip and deformation twins in the **α**_**2**_-Ti_3_Al and **γ**-TiAl crystals, which make the intragranular strength higher than the interface strength (including the grain boundary and lamellar interface). Therefore, the macro-plastic deformation is mainly coordinated by the boundary sliding and most of the internal stresses act on the interface^37^. As results, the crack propagation tends to along the interface causing the intergranular fracture (**L**_**5**_) and the trans-lamellar crack is prone to form terraced cleavage fracture (**L**_**6**_), as illustrated in Fig. 15(b). For the small precipitated particles (**B**_**2**_), the cracks could bypass forming branch crack (**L**_**7**_ and **L**_**8**_). Because of the weakness grain boundary, the compressive strength could not be improved by the grain refinement strengthening, while the compressive strain has been gradually increased with the ultrasonic vibration time by the large amount of grain boundary sliding.

### Experimental materials and methods

In this study, the basal as-cast ingot of Ti446Nb1Cr2V (nominal alloying composition, at.%) was prepared by vacuum induction melting at argon protective atmosphere and the ingot was remelted three times for homogenous composition. Small round bars with size of Ø20 × 50 mm^2^ were cutting from the basal ingot, which were remelted in the Y_2_O_3_ ceramic mould by high frequency electromagnetic induction heating. After cutting off the heating power, the molten melt was subjected to ultrasonic vibration for 15 s, 30 s, 45 s and 60 s, respectively. For comparison, the specimen without ultrasonic vibration (0 s) was also prepared under the same solidification conditions. The ultrasonic vibration was produced by a commercial ultrasound generator with a stainless steel radiator and the maximum ultrasonic power is 1200 W at the constant ultrasonic frequency of 20 kHz. In this study, the ultrasonic radiator was pressed on the top of ceramic mould and the ultrasonic vibration was conducted into the melt through the ceramic mould.

For microstructure observation, the specimens were prepared under the standard metallographic process. Optical microscopy (OM) and scanning electron microscopy in back-scattered electron mode (SEM-BSE) were used to investigate the microstructural characteristics after ultrasonic vibration. The chemical composition was analyzed by the energy disperse spectroscopy (EDS) equipped on the scanning electron microscopy. The phase constitution was identified by the X-ray diffractometer (XRD) with the scanning speed of 10 degrees per minute. The Vickers microhardness was measured by the digital microhardness tester under the press of 10 N for 10 s. The compression samples with the size of Ø4 × 6 mm^2^ were cutting from the same position of specimens by wire-electrode cutting and the testing samples were manual grinded before compression testing. The compression testing was conducted on the electron universal testing machines under the constant strain rate of 10^−4^ s^−1^ at the atmospheric temperature.

## Conclusions

1. Ultrasonic vibration could eliminate the shrinkage cavity and porosity obtaining a high quality casting ingot. The coarse microstructure is modified into fine globular grain structure with a homogenous element distribution and small precipitated particles.
2. Ultrasonic vibration could improve the phase ratio of **α**_**2**_-Ti_3_Al and **γ**-TiAl, weaken the effects of β-alloying elements on the equilibrium diagram and promote the composition and phase constitution more close to the equilibrium level.
3. Ultrasonic vibration is beneficial for the alleviation of element segregation and elimination of precipitated phases (**B**_**2**_), while the alloying element enrichment in the remnant small precipitated particles (**B**_**2**_) is aggravated leading to a higher alloying element segregation coefficient.
4. Under ultrasonic vibration, the large lamellar colony is refined from 534 μm to 56 μm and the prediction model for theoretic average grain size is established as 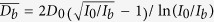 with a theoretic value of 64 μm for Ti44Al6Nb1Cr2V in this study.
5. As the increasing of ultrasonic vibration time, the microhardness, yield strength and compressive strain are gradually increased by 23.72%, 181.88% and 63.4% due to the fine grain strengthening, while the compressive strength is improved by 24.47% contributed by solution strengthening and the compressive strength retains almost constant as the ultrasonic vibration time.
6. The theoretical critical ultrasonic intensity for Ti44Al6Nb1Cr2V alloy is approximately 220 W cm^−2^ much higher than the light alloys; the ultrasonic grain refinement efficiency shows an exponential increasing as the ultrasonic vibration time, which shows a theoretic limit maximum value of ***E***_***lim***_ = 88%; the prominent grain refinement for TiAl-based alloys by ultrasonic vibration is the cavitation-enhanced nucleation rather than the cavitation-induced dendrite fragmentation.

## Additional Information

**How to cite this article**: Ruirun, C. *et al*. Effects of ultrasonic vibration on the microstructure and mechanical properties of high alloying TiAl. *Sci. Rep.* **7**, 41463; doi: 10.1038/srep41463 (2017).

**Publisher's note:** Springer Nature remains neutral with regard to jurisdictional claims in published maps and institutional affiliations.

## Figures and Tables

**Table 1:** Chemical compositions of matrix and precipitated phases (at.%).

Specimen	Phase	Ti	Al	Nb	Cr	V
0 s	Matrix	44.40	47.15	5.81	0.85	1.79
	B_2_	49.91	36.83	6.93	2.46	3.87
15 s	Matrix	44.95	46.44	5.89	0.91	1.81
	B_2_	48.72	36.43	7.05	3.13	4.67
30 s	Matrix	45.59	45.63	5.93	0.96	1.89
	B_2_	46.36	36.29	7.23	4.47	5.65
45 s	Matrix	46.06	44.94	6.01	1.02	1.97
	B_2_	45.46	36.18	7.64	4.71	6.01
60 s	Matrix	46.99	43.76	6.14	1.05	2.06
	B_2_	43.97	36.07	7.66	5.23	7.07
